# Neurological Neonatal Birth Injuries: A Literature Review

**DOI:** 10.7759/cureus.1938

**Published:** 2017-12-12

**Authors:** Naomi Ojumah, Rebecca C Ramdhan, Charlotte Wilson, Marios Loukas, Rod J Oskouian, R. Shane Tubbs

**Affiliations:** 1 SGU Department of Anatomical Sciences, Seattle Science Foundation; 2 Department of Anatomical Sciences, St. George's University School of Medicine, Grenada, West Indies; 3 Seattle Science Foundation; 4 Department of Anatomical Sciences, St. George's University School of Medicine, Grenada, West Indies; 5 Neurosurgery, Swedish Neuroscience Institute; 6 Neurosurgery, Seattle Science Foundation

**Keywords:** birth injury, paralysis, neonate, klumpke’s palsy, erb’s palsy, caput succedaneum, cephalohematoma, subgaleal hemorrhage

## Abstract

Birth injuries are a diverse set of traumas afflicting a newborn during labor and/or delivery. These range from temporary paralysis to hematomas. Herein, a comprehensive review of the birth injuries is presented, including the risk factors, classification of various paralyzes and nerve damage, as well as bleeding complications. The predicted outcomes and complications, as well as the treatment options for various birth injuries, are also discussed.

## Introduction and background

A birth injury is defined as structural damage or functional deterioration of a newborn secondary to a traumatic event that occurred during labor, delivery or both. Amniocentesis and intrauterine transfusions can cause injuries before birth, and therefore are not considered as birth injuries. The injuries following neonatal resuscitation procedures are also not classified as birth injuries. Some injuries can take place from use of fetal scalp electrodes and intrapartum heart rate monitoring and are characterized as birth injuries [1]. The neonatal birth injuries occur occasionally with an incidence of approximately 0.06-0.08% of live births [2] and account for less than 2% of the neonatal deaths [3]. These injuries frequently occur during the second stage of labor, in which the fetus descends through the birth canal [4].

## Review

Risk factors for birth trauma

There are many identifiable risk factors that increase the likelihood of neonatal birth injuries. These can be classified into three broad categories: fetal presentation, delivery mechanisms, and maternal factors. The fetal factors include macrosomia, breech presentation, abnormal fetal presentation, prematurity and precipitous delivery. Macrosomia results in a number of related injuries such as shoulder dystocia, rib fractures, clavicular fractures, cephalohematoma, and caput succedaneum. The poorly controlled maternal diabetes is one of the leading causes of macrosomia [1]. A birth weight of 4,000-4,500 g is associated with a twofold increase in the birth injury risk. This risk increases threefold if the birth weight is between 4,500 and 5,000 g, and more than 4.5-fold if the newborn weighs more than 5,000 g [1]. The breech presentation can result in brachial plexus palsies, intracranial hemorrhage, gluteal lacerations and long bone fractures. The rate of birth injuries for infants with breech presentation born by cesarean delivery without a trial of labor is 0.6%, 1% for cesarean delivery with labor, and 3% for the vaginal delivery [5]. An abnormal fetal presentation, such as transverse, compound, face, and brow can result in lacerations, excessive bruising, and retinal hemorrhage. Prematurity and precipitous deliveries are both related to bruising as well as intracranial and extracranial hemorrhage. The precipitous delivery can additionally cause retinal hemorrhaging [1].

Delivery mechanisms include obstetric instrumental techniques such as forceps delivery, vacuum extraction, or a combination of both, which increases the ease of descent but can potentially induce injury [4]. The vacuum extraction has a threefold increase, whereas forceps usage has a slightly higher fourfold increase in the birth injury risks when compared to unassisted vaginal deliveries [1]. There is a four to five times increase in the risk of cephalohematoma with forceps usage, an eight to nine times risk with vacuum-assisted delivery and 11-12 times increase with assistance from both the instruments [6].

Maternal factors include age, number of pregnancies, and pelvic anatomy. Extreme maternal ages (less than 16 and greater than 35 years), primigravida, cephalopelvic disproportion, short maternal stature, and maternal pelvic abnormalities predispose a fetus to birth injuries [1].

Obstetric brachial plexus injuries

Obstetric brachial plexus palsy is defined as a flaccid paresis of an arm at birth, with the passive range of motion being greater than the active range of motion [7]. The incidence rate of obstetric brachial plexus injury is approximately 0.15% in the United States [8], but can vary between 0.04-0.6% according to other reports [9]. Almost 80% of these injuries involve the cervical spine C5-C6 nerve roots (Erb-Duchenne palsy). The obstetric brachial plexus injuries tend to be transient with 70-95% of the cases resolving within a year. However, approximately 5-8% of the cases result in the persistent and permanent injuries [10-11]. The injuries that involve cervical spine- thoracic vertebrae C8-T1 nerve roots (Klumpke palsy) are more likely to persist with only 40% resolving within a year [12].

The patients with permanent obstetric brachial plexus injuries often develop bony asymmetry as a result of muscle imbalance on the developing bony elements of the infant’s shoulder [13]. The examples of these deformities include the internal rotator and adductor contractures, glenohumeral dysplasia, posterior humeral head subluxation or dislocation and scapular elevation and rotation. The surgery is usually needed to improve the limb function, otherwise, these patients may have long-term morbidity [9].

The birth weight among the injured patients tend to be higher than average, but it is not associated with injury severity. Nath, et al. [9] found that macrosomic fetuses frequently experience shoulder dystocia and develop permanent obstetric brachial plexus injuries despite the fact that macrosomia is said to be one of the primary indicators of the permanent injury. They concluded that a greater birth weight does not affect the prognosis of brachial plexus injury, as infants with birth weights that are normal for gestational age are still susceptible to severe permanent brachial plexus injuries. During delivery, maneuvers involving twisting and extension of the head can result in stretching of the neck and may be responsible for the obstetric brachial plexus injuries. According to Gonik, et al., lithotomy positioning during the delivery can greatly increase the brachial plexus stretch, whereas McRobert’s maneuver resulted in 53% less stretch. This was determined based on the computational modeling of intrauterine forces [14].

Brachial plexus injuries

The classic obstetric brachial plexus birth injury is Erb’s paralysis and is more common than Klumpke’s palsy and facial nerve injury. The injury can consist of two or more spinal roots. The degree of severity can vary from neuropraxia and axonotmesis to more serious injuries such as root avulsion [15]. The excessive widening of the angle between the head and the shoulder is the proposed mechanism for injury to the upper roots in the plexus (Erb’s palsy), while abduction and backward rotation damages lower roots (Klumpke’s palsy). Total plexus palsy (injury to C5 to T1) is also possible, although rare. The shoulder dystocia and vaginal breech delivery to a lesser extent, are strongly associated with the brachial plexus injuries; it has also been commonly associated with poor obstetric techniques [16]. Allen suggested a one-to-one correspondence between the shoulder dystocia and Erb’s palsy, especially in the cases with the permanent injury [17]. Other risk factors include macrosomia, multiparity, prior delivery of a child with obstetric brachial plexus palsy, breech delivery, shoulder dystocia, vacuum and forceps-assisted delivery, and excessive maternal weight gain [18]. The caesarian delivery is not free from implication either; out of 22 deliveries, one total brachial palsy occurred with the cesarean delivery [19].

The treatment is aimed at preventing contractures and can range from splinting and encouraging passive exercises in mild cases to the surgical exploration and grafting in more serious cases or for those who do not show recovery by three months [16, 20-21]. The nerve reconstruction is somewhat controversial; however, if there is no improvement at three months, root avulsion is suspected and these patients are eligible for the surgery [1]. Al-Al-Qattan, et al. observed good results with tendon transfer in the treatment of Erb’s palsy [22]. For patients presenting late (four-12 months) with poor or no recovery of elbow flexion, they underwent biceps brachii nerve transfer at the neck level (using intraplexus neurotization of the anterior division of the upper trunk). In patients older than 12 months, biceps neurotization was performed at the arm level using either a fascicle from the median or ulnar nerves. The motor assessment was done by the Toronto grading system and they noted good results in 90% of the cases.

Erb’s paralysis

Erb’s paralysis or Erb’s palsy occurs as a result of damage to the C5-C6 motor roots, in which the C5 and C6 unite to form the upper trunk of the brachial plexus [23]. The biceps brachii, brachialis, supinator, supraspinatus, and infraspinatus are affected. As a result, the classic presentation is adduction and medial rotation of the arm, and pronation of the forearm. The biceps reflex and sensation are also lost on the lateral arm. If the C7 nerve root is also involved, the wrist extensors are affected, resulting in wrist flexion and the classic waiter's tip posture [24]. Atrophy of the affected muscles occurs around the second year of the life [23]. There are variations to the presentation of Erb’s palsy and the extent of injury determines the prognosis. Chater, et al. [16] presented several cases; the first case was a 4398 g baby delivered vaginally using low forceps. The patient had shoulder dystocia, which led to a difficult extraction and Erb’s palsy was immediately noted with no deltoid function and a weak biceps brachii function, which was regained in five weeks with full recovery in three months. In the second case, a 4200 g baby was delivered with low vacuum suction, complicated by the moderate shoulder dystocia, which required suprapubic pressure, McRobert’s maneuver, and a “corkscrew” maneuver. Severe Erb’s palsy was immediately noted with little wrist extension and no bicep or deltoid function. In six weeks, there was still no improvement and the plastic surgery was advised if the dysfunction still persisted at five months. The third case was a 2216 g vaginal breech delivery, requiring forceps assistance. A left Erb’s palsy was noted with no contractions in triceps, biceps, and deltoids, but good power in all wrist movements. The recovery was marginal and at eight months, a magnetic resonance image (MRI) suggested avulsion of the cervical roots C5 and C6. At 8.5 months, neurolysis of C5 and C6 was performed with still no function at age six. The right deltoid and pectoralis major showed marked wasting, there was decreased strength in the triceps and biceps, and good grip with mildly decreased wrist extension. It was predicted that the patient might always be handicapped [16]. A 1948 study of 37 cases of Erb’s palsy was investigated which led to the following conclusions: the condition is slightly more common in males; injury to the supraclavicular portion is the cause of injury, therefore a skilled obstetric technique, especially in the use of forceps, should be employed; large babies, especially in primigravida are more at risk of injury; early treatment, specifically from birth, yields good results [20]. A delay in the treatment puts the formation of contractures at risk, causing the impaired function of the shoulder and elbow [20]. In contrast to these findings, a more recent study by Toopchizadeh, et al., which involved 42 children demonstrated that there were more female patients and the right arm was more commonly affected than the left arm [18].

When damage to C8 and T1 are included, there is total limb paralysis [24]. This is also known as the Erb-Klumpke paralysis. In about 5% of the patients, C3 and C4 can be injured (in addition to C5 and C6) leading to phrenic nerve dysfunction and paralysis of the hemidiaphragm [16]. An associated injury to the sympathetic trunk results in Horner’s syndrome (ptosis, miosis, and anhidrosis) and suggests a proximal T1 injury [24].

Klumpke’s paralysis

Klumpke’s paralysis involves C7, C8 and T1 nerve roots which results in the loss of function of the intrinsic muscles of the hand as well as the wrist and finger flexors [23-24]. The incidences are as low as 0.6% and some suggest the reason for this is modern obstetric practice and a sharp decline in the vaginal breech deliveries where there is a risk of hyperabduction of the arms [19]. Others also suggest that only a few reports exist in the literature because it may be a late presentation of a total brachial plexus injury in which the upper plexus functions have recovered [24]. In contrast to Erb’s palsy, which has a higher incidence, only approximately 40% of the Klumpke’s palsy cases are expected to resolve after one year, in contrast to the cases of the Erb’s palsy with up to 90% resolution by one year of life [12]. The studies have also found a higher incidence of the breech presentations in their reviews of the reported Klumpke’s palsy [22]. However, Buchanan, et al. presented an isolated case (upper plexus not involved) of left Klumpke’s palsy suggesting that the mechanism of the injury may not be hyperabduction [24]. The sympathetic trunk may also become injured in Klumpke’s palsy producing Horner’s syndrome, especially when T1 is involved [24].

Facial nerve paralysis

Facial nerve paralysis can result from perinatal trauma. The main reported risk factors associated with traumatic facial paralysis are primigravida, birth weight greater than 3500 g, forceps usage, cesarean birth, and prematurity. These cases typically have a good prognosis, with infants recovering without sequelae [25]. The mechanism of the injury is suggested to be from the pressure of the forceps blade on the facial nerve trunk as it leaves the stylomastoid foramen or as it crosses the ramus of the mandible. The injury may also be from the pressure on the promontory sacrum and ischium of the mother when the head is arrested at one these points. The first noticeable sign is a failure to close the affected eye and can result in a peripheral lesion affecting one half of the face. The majority of the infants with traumatic facial nerve palsy will recover within the first two months of life [23, 26].

Intracranial hemorrhage

Trauma during delivery is the main causes of intracranial hemorrhage. The risk factors include excessive molding of the head in prolonged labors, breech deliveries, and use of forceps [23]. Full-term infants with low birth weight and premature infants are at a greater risk of a hemorrhage occurring in utero due to hemodynamic instability [27]. Towner, et al. concluded that the rate of hemorrhages was higher in the infants delivered with the use of vacuum extraction, forceps or caesarian section during labor than in the infants delivered spontaneously or via caesarian before labor, suggesting abnormal labor as a common risk factor [28]. The excessive molding may cause rupture of the tentorium, resulting in a subdural hemorrhage [27]. The incidence of subdural hemorrhage in spontaneous deliveries is 0.003%, with incidences doubling with vacuum or forceps usage, and almost 10 times higher with the use of both vacuum and forceps [1]. Most of the bleeding is of venous origin from small vessels coursing along the tentorium such as the vein of Galen, which joins the straight sinus, and the transverse and straight sinuses or the internal cerebral veins [23]. Tearing of bridging blood vessels or the sinuses causes subarachnoid hemorrhage [27]. The hemorrhage may be limited to the surface of the cortex and meninges, but bleeding in the cerebral tissue and the ventricles is possible although less frequent in term newborns [23]. Intraparenchymal and intraventricular hemorrhage occurs at a much significantly lower rate in full-term newborns compared to premature newborns. This suggests an environmental contribution factor such as instrumental delivery, although a primary clotting abnormality or congenital vascular abnormality can also be responsible [29]. The intraventricular hemorrhage in a newborn commonly originates from the choroid plexus (cryptic hemangioma) or as an extension of the thalamic hemorrhage or subependymal germinal matrix bleed [30]. The epidural bleeds are rare because the middle meningeal artery is not yet encased within the bone and moves freely away from displacements of the skull. However, the epidural bleeds are still possible during a difficult forceps extraction, causing the outer layer of the dura to detach from the inner skull [27].

The clinical presentation depends on the extent of the bleed. Accumulation of blood in the posterior fossa can cause a tense or bulging fontanelle, which increases the head circumference and predisposes to central sleep apnea, bradycardia, and/or seizures. Stillborn births are a severe complication [23]. In cases with minimal trauma and less significant bleeding, the infant may show vague signs, suggesting that the brain is not the source of the problem. Therefore, because of only a small fraction of newborns present with the clinical symptoms, intracranial hemorrhage is probably higher than reported. The treatment is mainly prophylactic. The newborns with intracranial hemorrhage are treated in the intensive care unit with the aim of providing adequate ventilation, preventing metabolic acidosis, keeping vital organs well-perfused, and controlling seizure activity [27]. The retinal hemorrhage occurs in about 75% of the vacuum-assisted deliveries, 33% spontaneous vaginal deliveries and 6.7% of cesarean deliveries. The exact etiology is uncertain but due to the lowest incidence with cesarean deliveries, it has been suggested that retinal hemorrhages are caused by pressure exerted on the fetal head during passage through the birth canal [1]. A retinal hemorrhage is detected if a fundoscopy exam is done within the first 24 hours of life. These hemorrhages can be noted up to three to four weeks after birth and suspected birth trauma should not exclude investigating potential non-accidental causes. The patient has an increased risk of visual impairment if there is associated optic nerve injury.

Hematomas

Hematoma of the Sternocleidomastoid Muscle

The use of forceps in breech deliveries can cause the rupture of the sternocleidomastoid muscle (SCM) fibers and blood vessels, leading to a hematoma and subsequent development of a fibrous mass appearing at two to three weeks of age, resulting in a slight torticollis [23, 31]. The clinical features include tilting of the head to the affected side, chin rotated to the contralateral side, decreased active rotation to the affected side, positional plagiocephaly, and a tight SCM with a mass. Hypertropia may also be present on the contralateral side and may be a sign of a superior oblique palsy [32]. No treatment is necessary as the swelling usually disappears in two to three months [23].

Caput Succedaneum

Caput succedaneum is described as swelling that occurs due to the increased pressure of the vaginal and uterine walls on the fetal head during labor [33]. This pressure causes a hemorrhagic edema or a serosanguinous infiltration above the periosteum and below the skin or subcutaneous tissue [33]. The use of vacuum extraction and marked molding of the head also contributes to the swelling. The exact site of the fluid accumulation is the opposite site to which pressure is exerted. The continued pressure on one side of the head directly engaged in the pelvis in the first stage of labor allows for free circulation of blood and lymph through the tissues of the scalp and fascia. On the opposite side of the vertex, a portion of the scalp is under less pressure from the pelvis. It is here that blood and lymph are prevented from circulating through to the other side of the fetal head, allowing it to accumulate and consequently distend. The edema is soft and extends across the midline of the skull and crosses suture lines [34]. Since the swelling is directly under the skin, there can be overlying erythema, petechiae, and ecchymosis. The imaging should be considered in every child with a large caput succedaneum that fails to diminish in 48-72 hours if there is enlargement of the swelling more than 24 hours after delivery, and especially if there are neurological deficits or hemodynamic instability. Caput succedaneum tends to resolve in four to six days and the treatment is usually not necessary [1].

Cephalohematoma

Cephalohematoma is caused by a subperiosteal collection of blood due to rupture of vessels beneath the periosteum. The occurrence rate is 1-2% of all the deliveries regardless of the mode of delivery. Most cases are unilateral due to the fact that bleeding does not cross the suture lines as it occurs within a single cranial plate. Due to slow bleeding, the clinical presentation of swelling is delayed for several hours to days. A tense mass with ridge at the margin of the lesion and a recessed center is palpable. The lesion increases in size after birth and is usually largest on the third day of postnatal life [1]. The pathogenesis involves several factors such as the size of the skull and birth canal as well as the use of extraction instruments with the end result of the separation of the periosteum from bone [35]. Cephalohematomas tend to resolve at about three to four weeks. Occasionally, a palpable subcutaneous nodule may be felt due to calcification but this is resorbed during the next three to four months [1]. Osteomyelitis of the skull is a complication of cephalohematoma and Escherichia coli is the most common cause.

Subgaleal Hemorrhage

Subgaleal hemorrhage is a collection of blood between the epicranial aponeurosis and periosteum of the skull. The risk of the subgaleal hemorrhage is greater with vacuum extraction deliveries [34]. The subgaleal hemorrhage can cause sequestration of more than 40% of the newborn’s blood volume, which potentially results in the hemorrhagic shock. The mortality can be up to 14% from this hemorrhagic shock and its associated coagulopathy. The patients are managed with vital signs monitoring and serial measurements of both hematocrit and occipital frontal circumference (OFC) are performed. The subgaleal hemorrhage presents clinically with a classic triad of tachycardia, decreasing hematocrit, and increasing OFC during the first 24 to 48 hours of life. The palpation of the head shows bogginess of the subcutaneous tissue and possible fluctuance during the first 24 to 48 hours [1].

## Conclusions

It is important for the clinicians to understand the range of injuries that can occur to a newborn during birth. The fetal presentation, delivery mechanisms, and maternal factors can each influence the risk of a neonatal birth injury as well as the kind and severity of such injury. Such risk factors should be taken into consideration by obstetricians when dealing with complications during birth. Understanding the risk factors as well as the various classifications of the birth injuries ensures the best possible outcome for the newborn.
